# Cortisol regulates the paracrine action of macrophages by inducing vasoactive gene expression in endometrial cells

**DOI:** 10.1189/jlb.5A0215-061RR

**Published:** 2015-12-23

**Authors:** Uma Thiruchelvam, Jacqueline A. Maybin, Gregory M. Armstrong, Erin Greaves, Philippa T. K. Saunders, Hilary O. D. Critchley

**Affiliations:** * MRC Centre for Reproductive Health, The University of Edinburgh, The Queen’s Medical Research Institute, Edinburgh, United Kingdom; and; † MRC Centre for Inflammation Research, The University of Edinburgh, The Queen’s Medical Research Institute, Edinburgh, United Kingdom

**Keywords:** glucocorticoid receptor, progesterone, estradiol, angiogenesis, CXCL2

## Abstract

The human endometrium undergoes inflammation and tissue repair during menstruation. We hypothesized that the local availability of bioactive glucocorticoids plays an important role in immune cell–vascular cell interactions in endometrium during tissue repair at menstruation, acting either directly or indirectly via tissue resident macrophages. We sought to determine whether endometrial macrophages are direct targets for glucocorticoids; whether cortisol-treated macrophages have a paracrine effect on angiogenic gene expression by endometrial endothelial cells; and whether endometrial macrophages express angiogenic factors. Human endometrium (*n* = 41) was collected with ethical approval and subject consent. Donor peripheral blood monocyte-derived macrophages were treated with estradiol, progesterone, or cortisol. The effect of peripheral blood monocyte-derived macrophage secretory products on the expression of angiogenic RNAs by endothelial cells was examined. Immunofluorescence was used to examine localization in macrophages and other endometrial cell types across the menstrual cycle. Endometrial macrophages express the glucocorticoid receptor. In vitro culture with supernatants from cortisol-treated peripheral blood monocyte-derived macrophages resulted in altered endometrial endothelial cell expression of the angiogenic genes, *CXCL2*, *CXCL8*, *CTGF*, and *VEGFC*. These data highlight the importance of local cortisol in regulating paracrine actions of macrophages in the endometrium. *CXCL2* and *CXCL8* were detected in endometrial macrophages in situ. The expression of these factors was highest in the endometrium during the menstrual phase, consistent with these factors having a role in endometrial repair. Our data have indicated that activation of macrophages with glucocorticoids might have paracrine effects by increasing angiogenic factor expression by endometrial endothelial cells. This might reflect possible roles for macrophages in endometrial repair of the vascular bed after menstruation.

## Abstract (precis)

Cortisol regulation of macrophages induces angiogenic gene expression in endometrial cells at menstruation.

## Introduction

The endometrium is a complex multicellular steroid-target tissue that is repaired each month after menses without residual scarring or loss of function. Therefore, it provides an accessible in vivo human model of inflammation and efficient tissue repair. Tissue repair involves resolution of inflammation, angiogenesis, tissue remodeling, and formation of new tissue. Constituent cell types in the endometrium include stromal, epithelial, vascular, and immune cells. Dynamic cell-to-cell dialogue is essential to execute efficient endometrial shedding and subsequent re-epithelialization and stromal expansion, processes that are steroid regulated.

The ovarian steroid hormones estradiol and progesterone are well established as regulators of human endometrial function. The withdrawal of circulating estradiol and progesterone in the late secretory phase is associated with a striking influx of leukocytes, notably neutrophils and macrophages [1–3]. Macrophages are known to have a critical role in tissue repair in many tissues, including adult skin [4] and liver [5]. More than 15 yr ago, data were reported that provided evidence in support of a key role for endometrial macrophages in limiting the inflammatory response during endometrial shedding [6]. We recently reviewed the evidence that macrophages secrete factors that can influence endometrial repair [2]. Reciprocally, macrophage function can be influenced via endometrial cells, releasing factors such as M-CSF and GM-CSF [7, 8]. MMP-9, MMP-12, and MMP-14 and plasminogen activator instigate the breakdown of the endometrium at menstruation and are produced by endometrial macrophages premenstrually [9–11]. A recent study has also reported that an additional metalloproteinase, MMP-27, is expressed in CD45^+^/CD206^+^/CD163^+^ macrophages and that these cells were most abundant before menstruation [12]. Taken together, these results suggest that endometrial macrophages might play a role in both stimulating and restricting the inflammatory response during endometrial shedding. The role of macrophages during the resolution of menstruation has not been delineated.

In addition to ovarian-derived estradiol and progesterone, recent evidence has suggested that locally generated steroids, including estrogens [13] and glucocorticoids, might also play a significant role in endometrial function [14]. Locally produced glucocorticoids have been shown to limit inflammation in other tissue sites [15], mediated by the binding of cortisol to the nuclear GR and MR to exert its effects. In the endometrium, we have previously immunolocalized GRs to stromal, endothelial, and uterine NK cells [16]. The MR has been identified in glandular epithelial cells [17]. Previous studies have shown glucocorticoids to inhibit angiogenesis both in vitro and in vivo [18–21]. The enzyme 11β-HSD1 produces cortisol by the enzymatic reduction of cortisone; the reverse reaction is catalyzed by 11β-HSD2. Endometrial expression of the enzyme 11β-HSD1 has been reported to be upregulated at the time of menses, coincident with the maximal concentrations of GR mRNA in endometrial tissue homogenates [17]. Enhanced local inactivation of cortisol by 11β-HSD2 might be present in the endometrium of women with heavy menses [22], because the level of *HSD11B2* mRNA is 2.5-fold higher in these women than in healthy controls, predicting for substantially lower local endometrial cortisol concentrations. Tissue-resident human endometrial macrophages have been shown to express the β-isoform of the estrogen receptor [23]; however, to our knowledge, specific GR immunoreactivity in this immune cell type in the human endometrium has not been previously described.

We hypothesized that the local availability of bioactive glucocorticoids plays an important role in immune cell–vascular cell interactions in the human endometrium during tissue repair at menstruation. We demonstrate that endometrial macrophages express GRs and report a novel, macrophage-derived cortisol-dependent role in the regulation of angiogenesis within the endometrium.

## MATERIALS AND METHODS

### Patients and endometrial tissue samples

Endometrial biopsies (*n* = 41) were collected from women after written informed consent and local REC approval (REC approval code, LREC/07/S1103/29). Samples were collected from the uterine cavity using an endometrial suction curette (Pipelle, Laboratorie CCD, Paris, France) from women of reproductive age attending gynecologic outpatient departments across the National Health Service, Lothian, Scotland, United Kingdom. All women reported regular menstrual cycles (25–35 d) and no exogenous hormone exposure for 3 mo before biopsy. Women with known endometriosis and submucosal fibroids were excluded. The tissue was divided, fixed in neutral-buffered formalin for wax embedding, and placed in RNA-stabilizing reagent for PCR analysis. The biopsies were classified into phases of the menstrual cycle according to histologic dating [24], the reported last menstrual period, and the serum progesterone and estradiol concentrations at time of biopsy as measured by radioimmunoassay (**Table 1**).

**TABLE 1.** Details of endometrial biopsies used, including the day of the menstrual cycle and mean values for circulating estrogen and progesterone

Variable	Day of cycle of biopsy	Mean E2 (pmol/l)	Mean P4 (nmol/l)
Proliferative	3–17	339.8	3.2
Early secretory	10–21	462.6	54.6
Mid secretory	20–26	527.3	73.3
Late secretory	22–29	276.3	45.3
Menstrual	1–6	196.5	2.9

E2, estrogen; P4, progesterone.

### Dual immunofluorescence

Endometrial sections and positive control tissue sections (placenta, kidney, colon; 3-µm thick) were processed, as previously described [25]. In brief, the sections were exposed to xylene and rehydrated. Antigen retrieval (30 min, with increases to 126°C; 10 min, decreasing to 90°C, followed by a gradual cool down) was performed using sodium citrate (pH 6). Endogenous peroxidase activity was blocked with 3% H_2_O_2_. Normal goat serum was used as a protein block, and the sections were incubated with mouse monoclonal anti-CD68 (a pan-macrophage antigen; Dako, Glostrup, Denmark) at a 1:1000 dilution overnight at 4°C. Mouse IgG isotype was used as a negative control. Goat anti-mouse peroxidase secondary antibody (Abcam, Cambridge, United Kingdom) at a 1:500 dilution was applied for 30 min, followed by incubation with the TSA Cyanine 3 Tyramide System (Perkin Elmer, Waltham, MA, USA) for 10 min. The sections were microwaved with antigen retrieval buffer for 2 min (when subsequent antibodies raised in mouse were used) and incubated with appropriate normal serum (Supplemental Table 1) for 10 min, followed by the second primary antibody (Supplemental Table 1) overnight at 4°C. The sections were incubated with appropriate secondary antibodies (Supplemental Table 1) for 30 min, streptavidin Alexa Fluor 488 for 1 hr, followed by DAPI (Sigma-Aldrich, Dorset, United Kingdom) for 10 min. The sections were mounted with Permafluor (Thermo Scientific, Waltham, MA, USA) and analyzed using a Zeiss LSM710 confocal microscope system (Carl Zeiss, Jena, Germany).

### In vitro maturation of macrophage subtypes and generation of macrophage-conditioned media

Peripheral blood was obtained from consenting women taking the combined oral contraceptive pill (*n* = 9), hereafter described as donors, with local REC approval (REC approval no. 08/S1103/38). The blood was collected in 3.8% sodium citrate, and peripheral mononuclear blood cells were then isolated using a Percoll gradient. Monocytes were further separated by negative magnetic bead separation selecting for CD3-, CD7-, CD16-, CD19-, CD56-, CD123-, and glycophorin A-positive (Miltenyi Biotec, Cologne, Germany) cells, allowing elution of monocytes. The monocytes were cultured in RPMI 1640 medium (Sigma-Aldrich, St. Louis, MO, USA) with the addition of M-CSF (216.21 nM) for 4 d to differentiate the cells into macrophages. Macrophages were then treated with RPMI containing M-CSF alone (216.21 nM; M0) or with the addition of GM-CSF (285.71 nM) and IFN-γ (59.17 mM; M1), cortisol (1 μM), estradiol (10 nM), or progesterone (10 nM; M2) for an additional 48 h (Supplemental Table 2). Treatments to induce polarization of macrophages followed established protocols; macrophages stimulated with glucocorticoids and other steroids are grouped into the M2 or “nonclassic” phenotypic classification [26]. By stimulating macrophages with different steroids, we aimed to simulate the endocrine environment of the menstrual cycle. Thereafter, the cells were washed twice (to remove excess ligand) and resuspended in serum-free RPMI for 24 h, after which the supernatant was stored as conditioned media (see HEECs section).

### HEECs

The HEECs were a gift from Dr. Graciela Krikun (Yale University School of Medicine); the isolation of these cells has been previously described [27, 28]. HEECs were grown without serum for 48 h, and then treated with conditioned media from macrophage cultures (see above) for 24 h. Controls were treated with RPMI alone to determine the basal expression of angiogenic genes in HEECs.

### Angiogenesis array

Total RNA was extracted from HEECs treated with peripheral blood monocyte-derived macrophage-conditioned media (*n* = 3 donors) using an RNeasy Mini Kit (Qiagen Ltd, Sussex, United Kingdom) according to the manufacturer's instructions. Samples were treated for DNA contamination via DNA digestion during RNA purification. After extraction, RNA was quantified using a spectrophotometer (NanoDrop 1000, version 3.7; Thermo Scientific, Wilmington, DE). RNA samples were reverse transcribed using a cDNA synthesis kit (SuperScript VILO cDNA synthesis Kit and Master Mix; Invitrogen, Carlsbad, CA, USA) according to the manufacturer’s instructions. The thermal cycling conditions were 20 s at 25°C, 60 s at 42°C, and 5 s at 95°C. A TaqMan Low Density Human Angiogenesis Array (Applied Biosystems, Foster City, CA, USA) was used to analyze 94 genes involved in angiogenesis according to the manufacturer’s instructions and run on a TaqMan Low-Density Array 396 well block.

### Quantitative RT-PCR

To validate the array and to determine the angiogenic gene levels within the human endometrium, we performed complementary single-gene TaqMan quantitative RT-PCR analysis. In brief, a reaction mix was prepared containing TaqMan Supermix (5.5 mM MgCl_2_, 200 μM dATP, 200 μM dCTP, 200 μM dGTP, and 400 μM dUTP), ribosomal 18S primers/probe (Life Technologies, Carlsbad, CA, USA), and reaction-specific forward and reverse primers and probes (Universal Probe Library (Roche, Indianapolis, IN, USA) for *CXCL2, CXCL8, CTGF,* and *VEGFC* (Supplemental Table 3). A no-template control (with water instead of cDNA) was included on each plate. PCR was performed using an Applied Biosystems Prism 7900 instrument, and the results were analyzed in triplicate using Sequence Detector, version 2.3, and the 2ΔΔCt method [29]. Expression of target mRNA was normalized to RNA loading for each sample using 18S ribosomal RNA as an internal standard.

### Statistical analysis

For cell culture, mRNA results are expressed as the *x*-fold increase, where relative expression of mRNA after treatment was divided by the relative expression after vehicle treatment. Data are presented as box and whisker plots, the median is indicated, and the whiskers represent the minimum and maximum values. Significant differences among raw data (2ΔΔCt values) were determined using the Kruskal-Wallis nonparametric test with Dunn's multiple comparison post-test (Prism, version 4.02; GraphPad Software, Inc., San Diego, CA, USA). A value of *P* < 0.05 was considered significant.

## RESULTS

### Endometrial macrophages contain GRs

**Figure 1.**
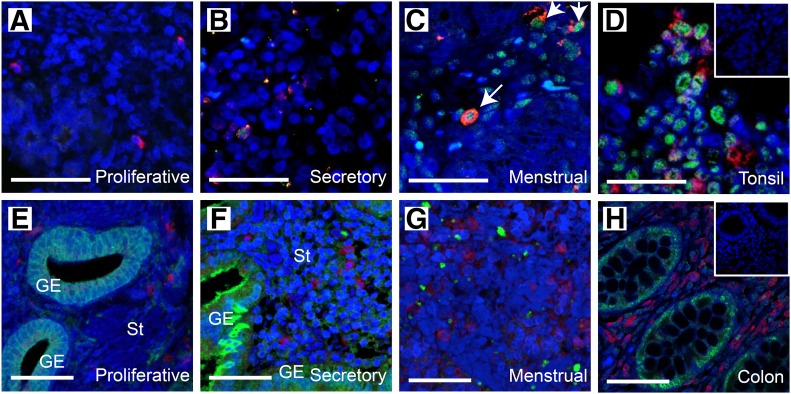
Endometrial macrophages contain GRs. Dual staining immunofluorescence revealed GRs (green) are present in endometrial macrophages (CD68^+^, red) during the secretory (B; *n* = 9) and menstrual (C; *n* = 3) phases but not in the proliferative phase (A; *n* = 5). Dual staining immunofluorescence revealed MRs (green) were not present in macrophages (CD68^+^, red) in endometrium during the proliferative (E), secretory (F), and menstrual (G) phases. Tonsil (D) and colon (H) were used as positive control tissues. (Inset) Negative control. Arrows highlight endometrial macrophages expressing GRs. Scale bars, 50 µm.

We performed immunohistochemical analysis to determine whether endometrial macrophages express GRs and MRs. Double immunofluorescence for CD68 and GRs revealed nuclear localization of GRs in CD68^+^ macrophages in both the secretory and the menstrual phases but not during the proliferative phase (**Fig. 1A–D**). The expression of MRs was detected in some endometrial cell types and in the positive control tissue (colon) but was not colocalized with CD68 macrophages (Fig. 1E–H), suggesting that the local increase in cortisol observed at menstruation has the potential to affect macrophage function via activation of GRs but not MRs.

### Cortisol-treated macrophages alter the angiogenic profile of HEECs

**Figure 2.**
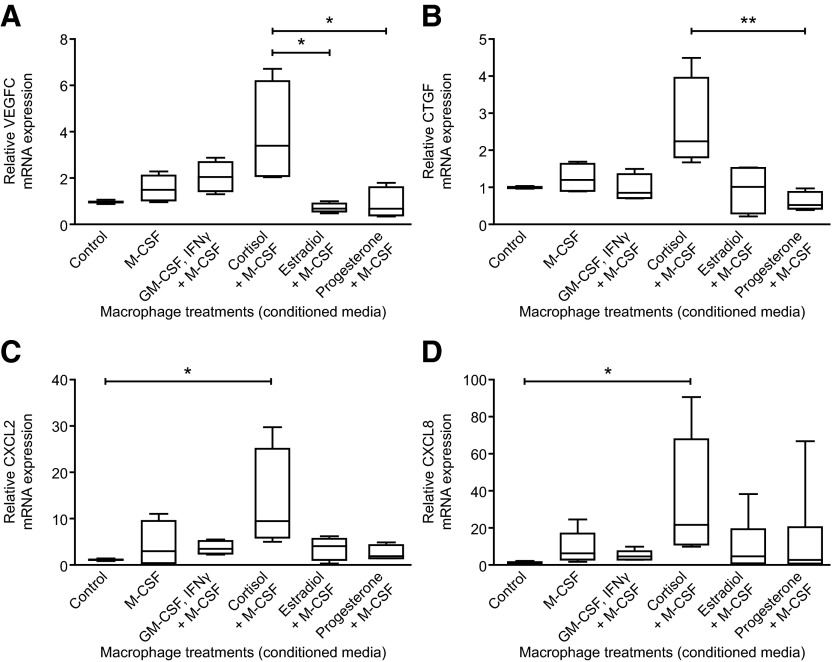
Treatment of macrophages with cortisol results in production of factors that have a paracrine impact on HEECs. mRNA concentrations in HEECs treated with conditioned medium from peripheral blood monocyte-derived macrophages cultured in the indicated conditions: (A) *VEGFC* (*n* = 4 values per treatment), (B) CTGF (*n* = 4), (C) CXCL2 (*n* = 4), and (D) CXCL8 (*n* = 5). *P ≤ 0.05, **P ≤ 0.01.

Peripheral blood monocytes were treated with M-CSF in order to stimulate maturation into macrophages. These macrophages were further treated with either M-CSF alone or media containing M-CSF and GM-CSF/IFN-γ, estradiol, progesterone, or cortisol for 48 h (Supplemental Table 2) using established protocols [30, 31]. They were then cultured for another 24 h with serum-free media to generate conditioned media, which was used to treat HEECs. Control HEECs were exposed to RPMI medium that had not been used to culture macrophages. Complementary DNA reverse transcribed from HEEC mRNA was used on a TaqMan Low-Density Array angiogenesis-specific array, which highlighted 69 genes that were altered by conditioned media from different macrophage subpopulations (Supplemental Fig. 1). Notably, stimulation by macrophage-conditioned media was lacking for many of the genes on the targeted array, with the exception of *CXCL2* and *CXCL8* (*IL-8*). We validated the expression of these 69 genes using individual gene specific assays and found that the concentrations of mRNAs encoded by *VEGFC, CTGF,* *CXCL2,* and *CXCL8* were all significantly altered by media from cortisol-treated macrophages (**Fig. 2A–D**), with no evidence of estradiol or progesterone eliciting a similar response to that of cortisol.

### Vascular remodeling factors are expressed by endometrial macrophages

**Figure 3.**
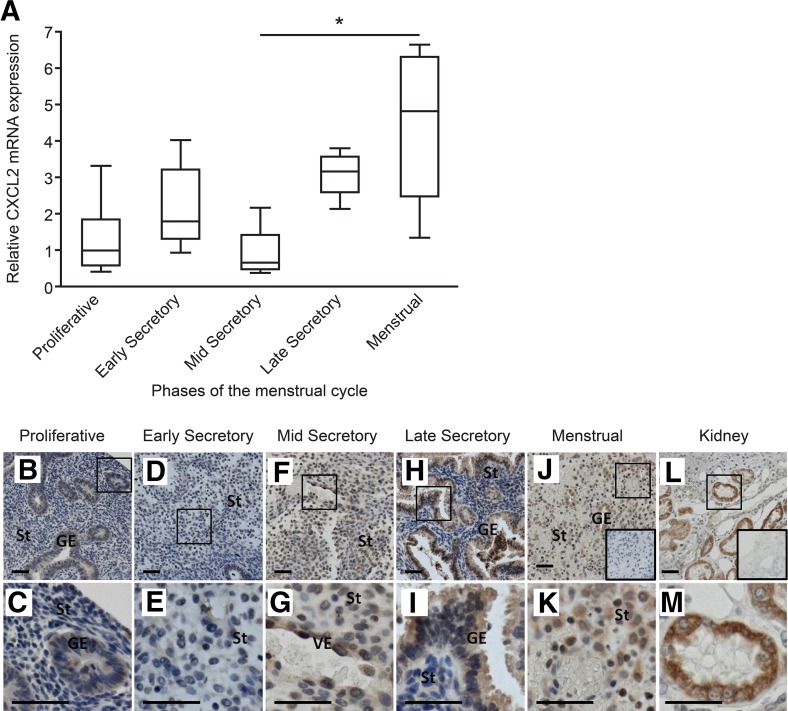
Expression of CXCL2 in human endometrial tissue is highest during the menstrual phase of the cycle. (A) The concentrations of CXCL2 mRNA in endometrial tissue homogenates were highest during the menstrual phase. Values are presented relative to the 18S ribosomal RNA endogenous control and to a placental sample as an internal control. (B and C) Proliferative (*n* = 5); (D and E) early secretory (*n* = 4); (F and G) mid-secretory (*n* = 4); (H and I) late secretory (*n* = 4); and (J and K) menstrual (*n* = 4; *P ≤ 0.05). Immunohistochemical staining for CXCL2 mirrored the results obtained for mRNA. Weak staining was observed during the proliferative (B and C; *n* = 5) and early secretory (D and E; *n* = 3) phases. Immunostaining appeared more intense as the cycle progressed into the mid-secretory (F and G; *n* = 3), late secretory (H and I; *n* = 3), and menstrual (J and K; *n* = 3) phases with clear immunopositive staining of decidualized stromal cells in the latter. Kidney (L and M) was used as a positive control. (Insets) Negative IgG isotype controls. Scale bars, 50 μm. GE, glandular epithelium; St, stromal compartment; VE, vascular endothelial cells. Higher magnification images (C, E, G, I, K, and M).

**Figure 4.**
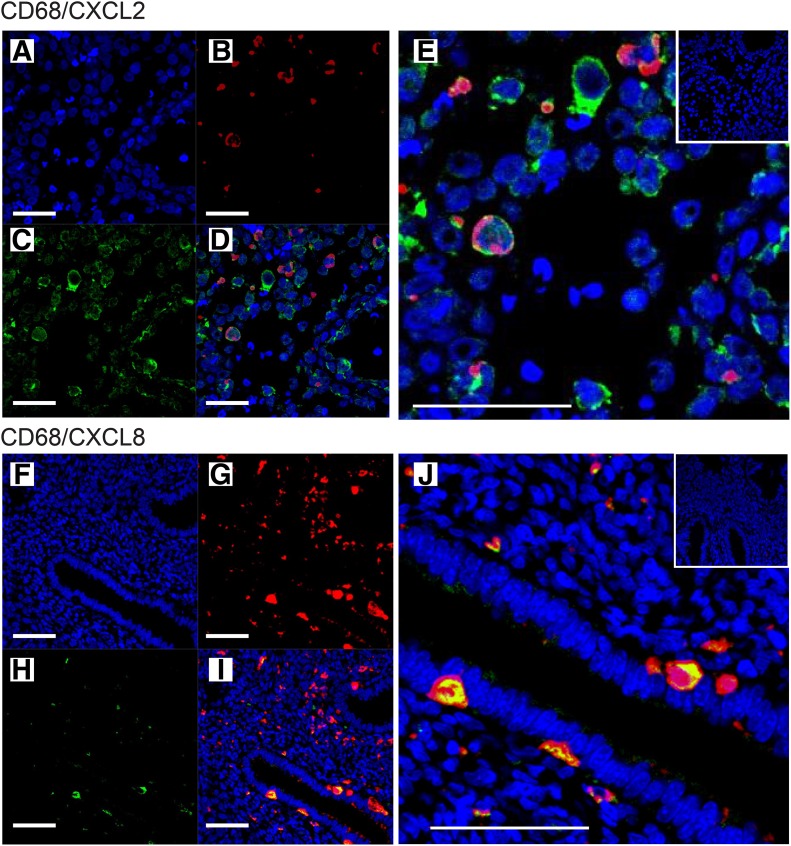
Immunolocalization revealed expression of vasoactive factors by human endometrial macrophages. Double immunofluorescent staining of endometrial macrophages for CD68 (red; B and G) and CXCL2 (green; C), or CXCL8 (green; H) revealed overlapping expression (D and I, respectively). Note that coexpression was particularly striking when individual cells were examined in enlarged/cropped images (E, CD68^+^/CXCL2^+^; J, CD68^+^/CXCL8^+^). (Insets) Negative IgG isotype controls. DAPI (blue) nuclear staining (A and F). Scale bars, 50 μm, *n* = 17.

Expression of CXCL2 has not been previously characterized in the human endometrium. We, therefore, extended our in vitro studies to examine its expression in our human endometrial tissue data set. Analysis of total RNA concentrations in endometrial tissue homogenates revealed maximal expression during the menstrual phase (**Fig. 3A**). Immunohistochemistry identified CXCL2 in the cytoplasm of glandular epithelial cells and endothelial cells (Fig. 3B–M), further supporting our gene expression data. Immunostaining for CXCL2 appeared most intense during the late secretory (Fig. 3H and I) and menstrual (Fig. 3J and K) phases compared with that in the tissues obtained during the proliferative (Fig. 3B and C), early secretory (Fig. 3D and E), and mid-secretory (Fig. 3F and G) phases. Double fluorescent immunohistochemistry demonstrated that both CXCL2 and CXCL8 are localized to endometrial macrophages at all stages of the menstrual cycle, underlining the potential for these cells to act as the source of these angiogenic factors (**Fig. 4A–J**).

## DISCUSSION

In the present study, we have shown that endometrial macrophages express the GR and display a phenotype consistent with a role in the regulation of endometrial angiogenesis. We found that culture supernatants from cortisol-treated macrophages stimulated changes in the concentrations of mRNA encoding angiogenic genes in endothelial cells. Our data provide new insights into the expression and regulation of the angiogenic factor CXCL2, revealing that its mRNA concentration was highest at menses. Also, the protein was present in multiple cell types, including endometrial endothelial cells and macrophages. Incubation of macrophages with cortisol resulted in the secretion of factors that stimulated increased expression of CXCL2 by endothelial cells, highlighting the potential for cortisol to act indirectly to change the gene expression of key angiogenic factors in the vasculature of the endometrium.

Progesterone withdrawal is the stimulus for menstruation; however, the progesterone receptor has not been identified in macrophages [32]. Therefore, macrophage function is considered to be only indirectly regulated by progesterone. We have demonstrated that endometrial macrophages express the GR. Cortisol acts via the GR and has numerous well-documented anti-inflammatory effects [33]. Within individual tissues, the glucocorticoid concentrations are regulated by the expression of the 11β-HSD enzymes, with 11β-HSD1 increasing local tissue availability of cortisol and 11β-HSD2 decreasing local tissue availability of this GR ligand. We have previously shown that endometrial HSD11B1 mRNA levels are significantly increased in endometrial tissue during menstruation, consistent with a role in the resolution and limitation of menstrual inflammation [17]. Because of the presence of GRs in endometrial macrophages, it is plausible that increased local cortisol levels have a direct effect on the function of macrophages, the numbers of which are increased during this phase of the menstrual cycle [2]. In vitro experiments treating peripheral blood monocyte-derived macrophages with synthetic glucocorticoids have shown the promotion of phagocytosis of apoptotic cells by macrophages [34].

Our gene expression studies involved in vitro treatment of donor peripheral blood-derived monocytes with cortisol, enabling us to model the menstrual events occurring in vivo. We acknowledge that these cells do not have an identical phenotype to endometrial tissue resident cells, but they were invaluable for our in vitro studies. We found that conditioned media from cortisol-exposed macrophages regulates the angiogenic profile of HEECs. This, in turn, suggests that exposure of macrophages to cortisol during the menstrual phase is likely to regulate angiogenesis via endothelial cells.

Cortisol has previously been shown to inhibit angiogenesis by the induction of anti-angiogenic gene expression [22]. It is highly likely that cortisol acts in a timely and cell-specific manner during the cycle to finely coordinate cell function. We suggest that cortisol can act directly on endometrial endothelial cells to inhibit angiogenesis and limit excessive bleeding, but it might also act on macrophages to promote endometrial repair and vascular bed replenishment. However, pro- and anti-angiogenic genes are thought to work in a synchronous balance [35], as evidenced previously in endometrial endothelial cells [36]. We proceeded to show that incubation of media containing products secreted by cortisol-treated macrophages induced changes in proangiogenic gene expression in endometrial endothelial cells. This finding is consistent with data showing that alternatively activated macrophages stimulate angiogenesis in endometriotic lesions [37]. One notable factor that we identified was CXCL2. CXCL2 is an important regulator of angiogenesis and thus is an attractive target when attempting to determine the angiogenic factors regulating the mechanisms involved in both breakdown and repair of blood vessels at menstruation. CXCL2 is a chemokine classically secreted by macrophages and is a potent chemoattractant to a number of immune cells such as polymorphonuclear leukocytes [38] and hematopoietic stem cells [39]. Regulation of the proangiogenic factor CXCL2 has been shown in tumor progression, vessel formation during tumor growth [40], and successful wound healing [41]. In the present study, we have described menstrual cycle stage-dependent expression of CXCL2 in endometrial homogenates, with highest expression occurring during menses. This is consistent with our previous published data of the expression of CXCL8 and CTGF [25, 42]. This maximal expression of angiogenic factors coincides with the time of endometrial repair and local angiogenesis.

Because the endometrial macrophage expresses the β-isoform of the estrogen receptor, its function might be influenced by estrogen during the proliferative and secretory phases. We have demonstrated that the effects of estradiol had less functional significance than those seen with cortisol pretreatment. It remains to be determined whether estradiol has a role in priming endometrial macrophages before cortisol exposure. Evidence has also shown that progestins, including progesterone, might mediate their actions via other nuclear receptors, including GRs [43, 44]; thus, further investigation into the context of macrophage activation in endometrium is warranted. Our findings have indicated that it is likely that increased local levels of cortisol at menstruation have an important role in regulating endometrial angiogenesis.

In conclusion, the data presented support a role for macrophages in endometrial function during menstruation and subsequent endometrial repair. Our data are consistent with the idea that local glucocorticoids regulate macrophage function in this complex reproductive tract tissue, which is also subject to exposure to other sex steroid hormones (estradiol and progesterone). These observations complement the published data regarding the other immune cells present in the human endometrium [45–47]. We believe our data provide evidence that cortisol-exposed macrophages play a role in menses and endometrial repair but recognize the need for future studies on the regulation of GR expression on endometrial macrophages. Ongoing work will delineate whether aberrant immune cell function during menstruation is involved in common menstrual disorders, such as heavy menstrual bleeding.

## AUTHORSHIP

U.T. contributed to the conception and design, acquired, analyzed and interpreted the data, and provided critical revisions to the manuscript. J.A.M. drafted the manuscript, contributed to data interpretation, and provided critical revisions to the manuscript. G.M.A. and E.G. acquired data and contributed to data interpretation and manuscript preparation. P.T.K.S. and H.O.D.C. contributed to the conception and design and provided critical revision of the intellectual content and text of the manuscript. All authors provided final approval of the version to be published.

## Supplementary Material

Supplemental Data

## Abbreviations

CD: cluster of differentiation
CTGF: connective tissue growth factor
GR: glucocorticoid receptor
HEEC: human endometrial endothelial cell
HESC: human endometrial stromal cell
HSD: hydroxysteroid dehydrogenase
MMP: matrix metalloproteinase
MR: mineralocorticoid receptor
REC: Research Ethics Committee
