# Supplementary material for: Cortisol regulates the paracrine action of macrophages by inducing vasoactive gene expression in endometrial cells
Source: J Leukoc Biol. 2015 Dec 23;99(6):1165–71. doi: 10.1189/jlb.5A0215-061RR (PMC4952012; doi:10.1189/jlb.5A0215-061RR)
Supplement: Supplemental Data [file supp_99_6_1165__index.html]

Cortisol regulates the paracrine action of macrophages by inducing vasoactive gene expression in endometrial cells — Cortisol regulates the paracrine action of macrophages by inducing vasoactive gene expression in endometrial cells — Cortisol regulates the paracrine action of macrophages by inducing vasoactive gene expression in endometrial cells — Supplemental Data 

# Cortisol regulates the paracrine action of macrophages by inducing vasoactive gene expression in endometrial cells

## Supplemental Data

- Supplemental Data
- Supplemental Data
- Supplemental Data
- Supplemental Data
