# Supplementary figures and images for: Cortisol regulates the paracrine action of macrophages by inducing vasoactive gene expression in endometrial cells
Source: J Leukoc Biol. 2015 Dec 23;99(6):1165–71. doi: 10.1189/jlb.5A0215-061RR (PMC4952012; doi:10.1189/jlb.5A0215-061RR)

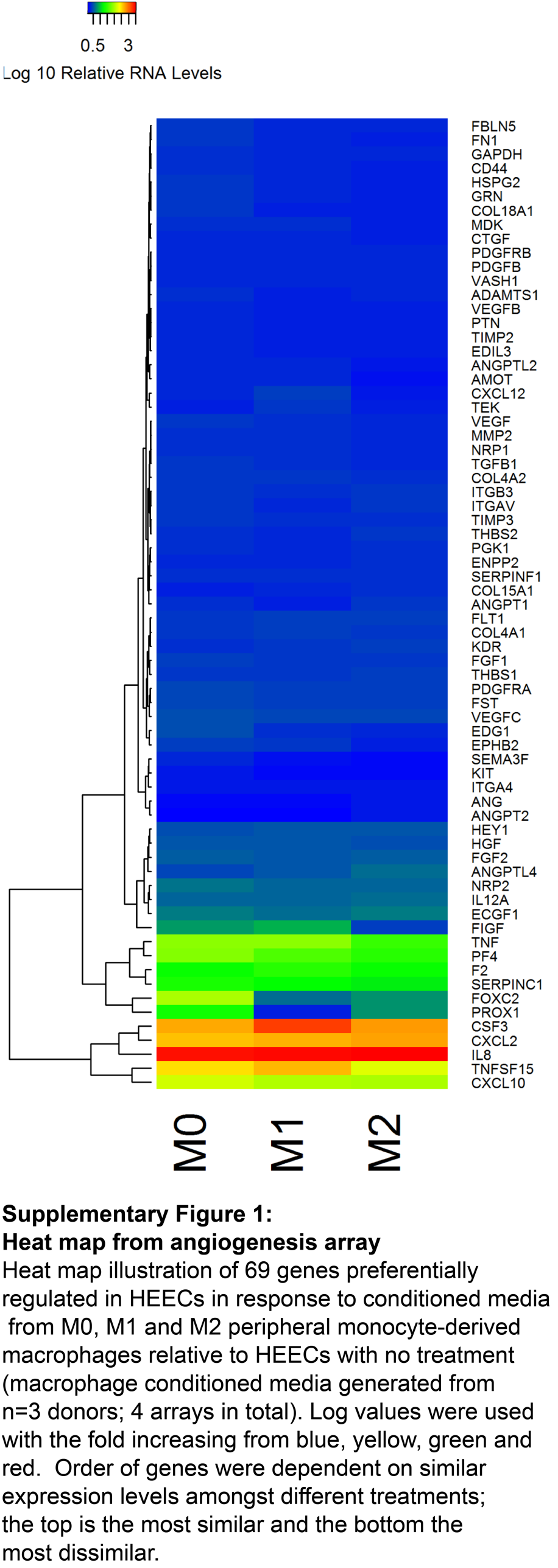

Supplement: Supplemental Data [file supp_jlb.5A0215-061RR_Supplemental_Figure1.tif]
