# Supplementary material for: Cortisol regulates the paracrine action of macrophages by inducing vasoactive gene expression in endometrial cells
Source: J Leukoc Biol. 2015 Dec 23;99(6):1165–71. doi: 10.1189/jlb.5A0215-061RR (PMC4952012; doi:10.1189/jlb.5A0215-061RR)
Supplement: Supplemental Data [file supp_jlb.5A0215-061RR_Supplemental_Table1.docx]

**Supplementary Table 1:** Antibodies used for immunohistochemical studies

| **Primary Antibody** | **Concentration** | **Species Raised** | **Supplier** | **Secondary Antibody** | **Negative control** |
| --- | --- | --- | --- | --- | --- |
| CD68 | 0.1 µg/mL | Mouse monoclonal | Dako (M0814) | Goat anti-mouse peroxidase (P0447, Dako) | Mouse IgG1 (MAB002, R&D) |
| Glucocorticoid Receptor (GR) | 0.8 µg/mL | Mouse monoclonal | Abcam (ab9568) | Goat anti-mouse biotinylated (BA9200, Vector) | Mouse IgG1 |
| Mineralocorticoid Receptor (MR) | 1:100 | Mouse monoclonal | Kind gift from Gomez-Sanchez (MRN-2-2D6) | Goat anti-mouse biotinylated | MR peptide (sc6860P, Santa Cruz;1:50) absorbed antibody |
| Interleukin 8 (CXCL-8) | 1:750 | Rabbit polyclonal | Lab bleed | Goat anti-rabbit biotinylated (BA1000, Vector) | Rabbit IgG (E0354, Dako) |
| CXCL2 | 0.5 µg/mL | Rabbit polyclonal | AbD Serotec (AHP773) | Goat anti-rabbit biotinylated | Rabbit IgG |
