# Supplementary material for: Cortisol regulates the paracrine action of macrophages by inducing vasoactive gene expression in endometrial cells
Source: J Leukoc Biol. 2015 Dec 23;99(6):1165–71. doi: 10.1189/jlb.5A0215-061RR (PMC4952012; doi:10.1189/jlb.5A0215-061RR)
Supplement: Supplemental Data [file supp_jlb.5A0215-061RR_Supplemental_Table2.docx]

**Supplementary Table 2:**

Treatments used for *in vitro* differentiation of donor peripheral blood monocytes into macrophage subtypes.

| **Polarization** | **Treatment** | **4 days** | **48 hours** |
| --- | --- | --- | --- |
| **M0** | **M-CSF** | M-CSF (216.2 nM) | M-CSF (216.2 nM) |
| **M1** | **GM-CSF and IFNγ** | M-CSF (216.2 nM) | M-CSF (216.2 nM),  GM-CSF (285.7 nM) + IFNγ (59.17 mM) |
| **M2** | **Cortisol** | M-CSF (216.2 nM) | M-CSF (216.2 nM) and Cortisol (1 μM) |
| **M2** | **Estradiol** | M-CSF (216.2 nM) | M-CSF (216.2 nM) and Estradiol (10 nM) |
| **M2** | **Progesterone** | M-CSF (216.2 nM) | M-CSF (216.2 nM) and Progesterone (10 nM) |
