# Supplementary material for: Cortisol regulates the paracrine action of macrophages by inducing vasoactive gene expression in endometrial cells
Source: J Leukoc Biol. 2015 Dec 23;99(6):1165–71. doi: 10.1189/jlb.5A0215-061RR (PMC4952012; doi:10.1189/jlb.5A0215-061RR)
Supplement: Supplemental Data [file supp_jlb.5A0215-061RR_Supplemental_Table3.docx]

**Supplementary Table 3 - PCR primers and probes**

**a) Roche probes**

| **Gene name** | **Forward Primer** | **Reverse Primer** | **Probe number (UPL)** |
| --- | --- | --- | --- |
| CXCL2 | cccatggttaagaaaatcatcg | cttcaggaacagccaccaat | 69 |
| CXCL10 | gaaagcagttagcaaggaaagg | gacatatactccatgtagggaagtga | 34 |

**b) Eurogentec probes**

| **Gene name** | **Forward Primer** | **Reverse Primer** | **Eurogentec Probe sequence** |
| --- | --- | --- | --- |
| CTGF | tgcaccgccaaagatggt | ggcacgtgcactggtacttg | ctccctgcatcttcggtggtacggt |
| CXCL8 | ctggccgtggctctcttg | ttagcactccttggaaaactg | ccttcctgatttctgcagctctgtgtgaa |
